# The new strategies to overcome challenges in protein production in bacteria

**DOI:** 10.1111/1751-7915.13338

**Published:** 2018-11-28

**Authors:** Anna Lipońska, Farès Ousalem, Daniel P. Aalberts, John F. Hunt, Grégory Boël

**Affiliations:** ^1^ Institut de Biologie Physico‐Chimique Sorbonne Paris Cité UMR 8261 CNRS‐University Paris Diderot 13 rue Pierre et Marie Curie 75005 Paris France; ^2^ Physics Department Williams College Williamstown MA 01267 USA; ^3^ Department of Biological Sciences Columbia University New York NY 10027 USA

## Abstract

Recombinant proteins are essential for biotechnology. Here we review some of the key points for improving the production of heterologous proteins, and what can be the future of the field.

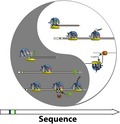

## Introduction

Protein production has been of great interest to industry for a long time: first for the food industry and household products, then bio‐production, now for medicine and biotech, tomorrow for the development of synthetic biology and protein nanomachines. Nowadays, market demand for proteins not only concerns chemical and food industries, but also pharmaceuticals (Palomares *et al*., 2002). From the time of the first commercialized pharmaceutical recombinant protein, human insulin (Gentech/Eli Lilly in 1982), the protein therapeutics market has been steadily increasing. From 2011 to 2016, 62 new biologics were approved by the FDA (Lagassé *et al*., 2017). Today, this production is centralized and large‐scale, but in the future, small‐scale manufacturing adapted to individual needs of smaller patient populations may become the standard (Crowell *et al*., 2018). An aim of the biological revolution will be to produce functional protein in a cost‐efficient manner.

In past decades, most proteins were extracted from the living organisms that produce them. This process was time‐consuming and resulted in low quantities of desired proteins. Along with science and biotechnology development, this problem was solved by heterologous protein overproduction in model organisms. The gene encoding the protein of interest is over‐expressed in another organism than the native one, such as in bacteria, yeasts, insect and human cell lines, each with advantages and disadvantages. The bacteria *Escherichia coli* is widely used because it is less time‐consuming and often more cost‐efficient than other systems, moreover it benefits from all the knowledge, genetic tools and new methods of protein production optimization.

Protein expression is a complex task; the whole process from transcription to translation involves hundreds of components and many variables that are cross‐correlated. Consequently, the optimization of the production can be performed by influencing different stages and changing different parameters. For the purpose of this short review, we focus mainly on optimization directly related to translation. We have divided this discussion into cis and trans‐optimization. Cis‐optimization concentrates on nucleotide sequence improvement whereas trans‐optimization will focus on the use of the right, optimized bacterial strain.

## Cis‐optimization

Sequence optimization consists of designing a DNA sequence that is optimal for expressing a protein. The DNA sequence is transcribed into mRNA, which is the template for protein synthesis catalysed by the ribosome. The synthesis of the protein starts with the binding of the small subunit of the ribosome upstream of the coding sequence at the Shine and Dalgarno (SD) sequence. This initiation can be modulated by modifying the SD sequence complementarity with the ribosomal anti‐SD sequence and its distance from the start codon (Schurr *et al*., 1993; Chen *et al*., 1994). The Salis group has developed an algorithm to optimize the SD site (Espah Borujeni *et al*., 2014).

### Choosing the right codons – frequent doesn't mean better!

The ribosome reads the mRNA sequence in three base groups called codons. The first codon read is the initiator codon, of which AUG is the most efficient of three possibilities. Each codon encodes an amino acid with the exception of the three stop codons that signal the end of the message. It is important to remember that we have 61 codons and only 20 amino acids, so most amino acids are encoded by more than one codon (called synonymous codons). The frequencies of use for each codon are not equal, some of them occur more often (frequent codons), whereas others rarely (rare codons). Since in *E. coli* the most frequent codons are decoded by the most abundant tRNAs, this codon usage is considered to correlate with the availability of some tRNAs, the most limiting step in translation elongation (Ikemura, 1981). Logically, it has been postulated that rare codons translate slower and therefore reduce protein production.

Native *E. coli* proteins that are highly expressed often use frequent codons. Hence, codon metrics based on codon frequency have been used for optimization of poor genes with low expression. Ikemura calculated the frequency of optimal codons in a gene, but the most widely used metric was a Codon Adaptation Index (CAI), also based on codon usage (Sharp and Li, 1987). These conventional observations led to the concept that the more frequent codons are the ‘good’ ones whereas the rare codons the ‘bad’ ones.

Optimization based on codon usage became routine, but its success has been variable, suggesting that it is not a rational optimization method. Indeed, several studies have recently shown that rare codons are not systematically correlated with low expression (Goodman *et al*., 2013; Boël *et al*., 2016). The concept that tRNA concentration controls elongation speed under normal physiological conditions has been challenged by a variety of different sources. The failure to observe a significant global correlation with ribosome dwell time and tRNA concentration in any prokaryotic ribosome profiling experiment (Mohammad *et al*., 2016; Aalberts *et al*., 2017) could reflect technical limitations in those methods, but their failure to provide support for the traditional model resonates with the failure to observe significant correlations in a variety of other global profiling studies conducted using orthogonal methods (Goodman *et al*., 2013; Boël *et al*., 2016). Overexpression could create stress on the tRNA pool that makes cognate tRNA concentration important under those conditions (Makrides, 1996), but there is no evidence of a systematic correlation even for expression because codons with similarly low frequency and cognate tRNA concentration have divergent influences on protein overexpression level (Boël *et al*., 2016).

**Figure 1:**
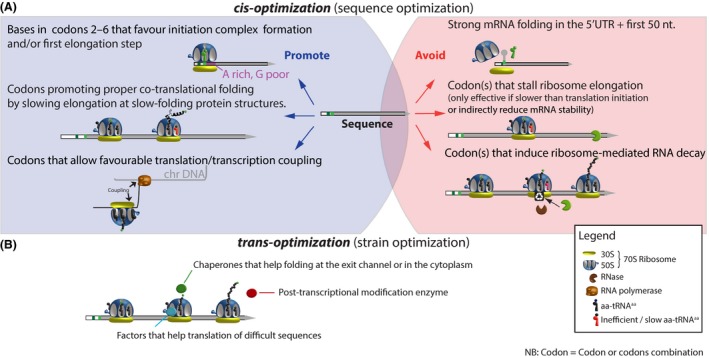
Strategies to optimize protein production. A. Factors to promote or to avoid for increased protein expression. B. Cellular factors that can help specific proteins to be properly synthesized.

We now see some intricate relations between codon usage and other central pathways like protein folding, mRNA degradation and transcription/translation coupling. Comprehension of those relationships will guide us towards more rational optimization strategies (Fig. 1A).

Some codons or codon combinations can stall ribosomes and thus reduce protein synthesis. This reduction can be exacerbated by the fact that stalled ribosomes can expose mRNA to RNases or/and actively recruit the RNase machinery (Fig. 1A) (Boël *et al*., 2016; Hanson and Coller, 2017).

Codon usage may also influence protein folding introducing a context dependency to codon choice. A change to a synonymous ‘faster’ codon, which locally speeds up translation, may allow the nascent peptide chain to rapidly and negatively influence protein folding (Komar *et al*., 1999). The use of some specific codons or codon combinations that slow the elongation process may allow pauses for the proper folding of the protein to occur. Sequence optimization based on harmonization of the codon frequency usage of the expression host to match the frequency used in the native host helps protein folding (Siller *et al*., 2010; Buhr *et al*., 2016). An evolutionist view of codon usage also shows that rare codons can be used to direct tRNA specificity during translation. Some rare codons are less prone to error than the frequent ones; therefore, they are more used to encode key amino acids of the protein (Drummond and Wilke, 2008). The challenge is to take all those parameters in account to generate the best sequence. The future will possibly be tailored optimization methods that account for protein specificity.

### Choosing the right codons – mRNA folding and base composition effects

mRNA secondary structures in the 5′ untranslated transcribed region (UTR) of the mRNA and the beginning of the coding sequence strongly influence gene expression. Folding of the mRNA can prevent the binding of the ribosome small subunit to the SD (Geissmann *et al*., 2009). Limiting the folding of this part of the mRNA is crucial for good sequence optimization. It has been shown that a higher amount of adenosine (A) in the first 18 nucleotides of the coding sequence increases the probability of higher protein expression, whereas G decreases it (U has an intermediate positive and C intermediate negative effect) (Boël *et al*., 2016). A synonymous codon substitution makes many changes simultaneously: the codon usage frequency, the base composition, the mRNA folding. All have a strong impact on translation. With this taken into account, we have to use more accurate tools for sequence optimization, one that can integrate multi‐parameter optimization.

### Transcription/translation coupling

In most biotechnological applications, protein expression in *E. coli* occurs by use of T7/IPTG system. IPTG induces synthesis of bacteriophage T7 RNA polymerase, which then can recognize the T7 promotor controlling expression of the desired protein. The T7 RNA polymerase is much faster than *E. coli* RNA polymerase; these kinetic differences limit the coupling of the translation with the transcription. Therefore, T7 RNA polymerase activity results in a mass production of mRNA, that is not protected by transcribing ribosomes, which occurs normally with the *E. coli* RNA polymerase (Iost and Dreyfus, 1995). Evolution of sequence optimization has to take those parameters into account. It is possible that the coupling with the RNA polymerase can be improved algorithmically in the future. In the case of the T7 expression, the best optimization may differ from the one used for *E. coli* endogenous RNA polymerase.

## Trans‐optimization

As discussed, cis‐optimization methods can help expression of proteins, but to get the best results, these should be combined with trans‐optimization methods. Optimization of growth media and temperature, the right concentration of inducer or use of protein fusion can play a big role as well. However, selecting of the right bacterial strain is particularly important to get the best results. When dealing with a protein prone to misfolding and aggregation, like membrane proteins, a strain co‐expressing molecular chaperones can be used (Fig. 1B).

Proteins with disulphide bonds are difficult to express because bacterial cytoplasm is typically not suitable for sulphide bond formation; however, *E. coli* strains have been successfully engineered to oxidize cysteines in the cytoplasm (Anton *et al*., 2016). Moreover, there are now *E. coli* strains that can perform post‐translational modifications like N‐glycolyzation, a modification generally occurring only in eukaryotic cells (Wacker *et al*., 2002) or acetylation (Johnson *et al*., 2010). These strains co‐express heterologous enzymes that can catalyse those modifications. Another challenge is the expression of membrane proteins which can create toxicity during their overexpression and can be misfolded. This effect can be reduced by using *E. coli* strains that use a more reduced T7 RNA expression than regular ones (Angius *et al*., 2018).

Recently identified translation factors assist the synthesis of sequences difficult to translate; for example, the factor Ef‐P, which suppresses translation inhibition at poly‐Proline stretches (Ude *et al*., 2013). These factors and others that remain to be discovered can be over‐expressed in specific strains to assist the synthesis of proteins that require their help. It is important to note that some trans‐optimization could change the influence of synonymous codons, making it possible that cis‐optimization and trans‐optimization cannot be done independently.

The future of optimization will integrate all those parameters and will fine‐tune them according to the nature of the protein to be synthesized. Translation speed will be encoded to facilitate protein folding, localization and post‐translational modifications. This will be coupled with an expression strain adapted for the specific protein.
